# Comprehensive review of the application of MP and the potential for graft modification

**DOI:** 10.3389/frtra.2023.1163539

**Published:** 2023-05-16

**Authors:** Paola A. Vargas, Christine Yu, Nicolas Goldaracena

**Affiliations:** ^1^ Division of Transplant Surgery, Department of Surgery, University of Virginia Health System, Charlottesville, VA, United States; ^2^ Department of Public Health Sciences, University of Virginia, Charlottesville, VA, United States

**Keywords:** machine perfusion, *ex vivo*, liver transplantation, marginal organs, donor pool

## Abstract

**Introduction:**

Following procurement, the liver graft is exposed to an ischemic period that triggers several pathophysiologic changes in response to oxygen deprivation. Therefore, the goal during organ preservation is to attenuate such response and provide an adequate environment that prepares the graft for its metabolic reactivation following implantation. This has been widely achieved via static cold storage preservation, where the maintenance of the graft using cold preservation solutions reduce its metabolic activity and confer cytoprotection until transplantation. However, despite being the gold standard for organ preservation, static cold storage holds several disadvantages. In addition, the ongoing organ shortage has led to the use of unconventional grafts that could benefit from therapies pre-transplant. Organ preservation via machine perfusion systems appears as a promising solution to address both.

**Methods:**

Here, we aim to present a state-of-the-art narrative review regarding liver graft modification options using machine perfusion systems in combination with adjuvant strategies including immunomodulation, gene therapy and pharmacotherapy.

**Results:**

Available reports are scarce and mostly on experimental animal models. Most of the literature reflects the use of normothermic or subnormothermic machine perfusion devices given that these particular type of machine allows for a metabolically active organ, and therefore facilitates its modification. Although limited, promising findings in available reports suggest that organ preservation using machine perfusion system when combined with alternative therapies can be feasible and safe strategies for graft modification.

**Discussion:**

Further research on clinical settings are needed to better elucidate the true effect of graft modification pre-transplant on short- and long-term graft and patient survival. There is a long way ahead to develop guidelines and approve these novel therapies for clinical practice. However, the path looks promising.

## Introduction

Despite having a futuristic ring to it, the concept of organ preservation using machine perfusion (MP) systems is far from modern. In fact, Belzer et al. performed the first successful human transplant using a machine-perfused graft more than half a century ago (1). The procedure took place 1 year after successfully achieving organ viability following *ex vivo* perfusion of canine kidneys in 1967 (2). The team decided to use the model for transplantation of a kidney graft in a 47-year-old man with a history of amyloidosis without a chance of alternative treatment (1). The authors’ promising results were then supported by many other studies, demonstrating MP superiority against static cold storage (SCS) in different areas, including a lower incidence of delayed graft function and improved graft survival (3, 4). Technical and logistics advances over the years have since allowed MP to become one of the key preservation methods for kidney transplantation (3, 4). Moreover, its clinical applications have reached different areas in transplantation, including organ preservation for lung, heart, and liver transplantations (LT).

Regardless of being around for many years, it was only in 2010 that the first clinical trial using MP for human liver grafts was published (5). Over a span of 4 years, 20 liver grafts from donors after brain death (DBD) were preserved from 3–7 h using a hypothermic machine perfusion (HMP) device (5). In the clinical trial, HMP was demonstrated to be a safe and feasible option, with potential superiority to SCS in terms of liver injury and postoperative complications (5). Since then, several experimental studies, along with a handful of clinical trials, have aimed to demonstrate the superiority of dynamic preservation methods—MP systems—in the reduction of ischemia reperfusion injury of the liver graft when compared to SCS (6–9). Thus, under the premise of reducing graft injury before transplantation, research efforts have been toward the evaluation of the potential role of MP as a pathway to lessen the unfavorable shortage of liver organs by increasing the usage of all available organs.

Hence, subsequent randomized and non-randomized clinical trials have included grafts that are often discarded due to poor quality, such as those from donors after cardiac death (DCD) and extended criteria donors (ECD) (Supplementary Table S1). Similarly, research involving the development and implementation of innovative techniques, such as hypothermic oxygenated perfusion (HOPE), dual-HOPE, and normothermic machine perfusion (NMP), have been on the rise (10–20). However, the potential benefits and superiority of MP not only encompass ischemic-related injury, but an umbrella of possibilities, including graft viability assessment and graft modification, which SCS is unable to offer. Here, we aim to present a state-of-the-art review regarding liver graft modification options using MP systems.

## Importance of graft preservation and overview of dynamic preservation techniques

After procurement, the liver graft is exposed to an ischemic period known as cold ischemia. During this period, the ischemic insult triggers several pathophysiologic changes in response to oxygen deprivation. Initial changes include the activation of Kupffer cells and sinusoidal endothelial cells, leading to a complex immunologic response that potentiates hepatocellular damage (21). In addition, hepatic ischemia leads to a detrimental metabolic cascade preceded by a decrease in oxidative phosphorylation, leading to ATP depletion, an increase in anaerobic metabolism, hepatocyte acidosis, dysregulation of ATP-dependent ion exchange mechanisms, mitochondrial dysfunction, cellular swelling, and death (21, 22). Moreover, damage to the sinusoidal endothelial cells also leads to damage of hepatic microcirculation—moderated by, among others, the release of thromboxane A2, which plays an important role in liver dysfunction after implantation (23). Although this complex process is still not fully elucidated, it is known that hepatic ischemia is a key determinant in the development of early allograft dysfunction and negative post-transplant outcomes (24).

Therefore, the goal during organ preservation is to attenuate the intense hepatic response to ischemia and to provide an adequate environment that prepares the graft for its metabolic reactivation after implantation (24). This has been widely achieved via SCS to reduce the graft metabolic activity along with the use of different hypothermic solutions to confer cytoprotection (25). However, despite being the gold standard for organ preservation, static cold storage holds several disadvantages, including a prolonged cold ischemia time and the inability to assess graft viability before implantation (24, 25). Hence, in an attempt to tackle the shortcomings of SCS, in summation with the need of more suitable liver grafts for transplantation, dynamic preservation techniques using a machine perfusion device came into sight as a promising option.

**Figure 1:**
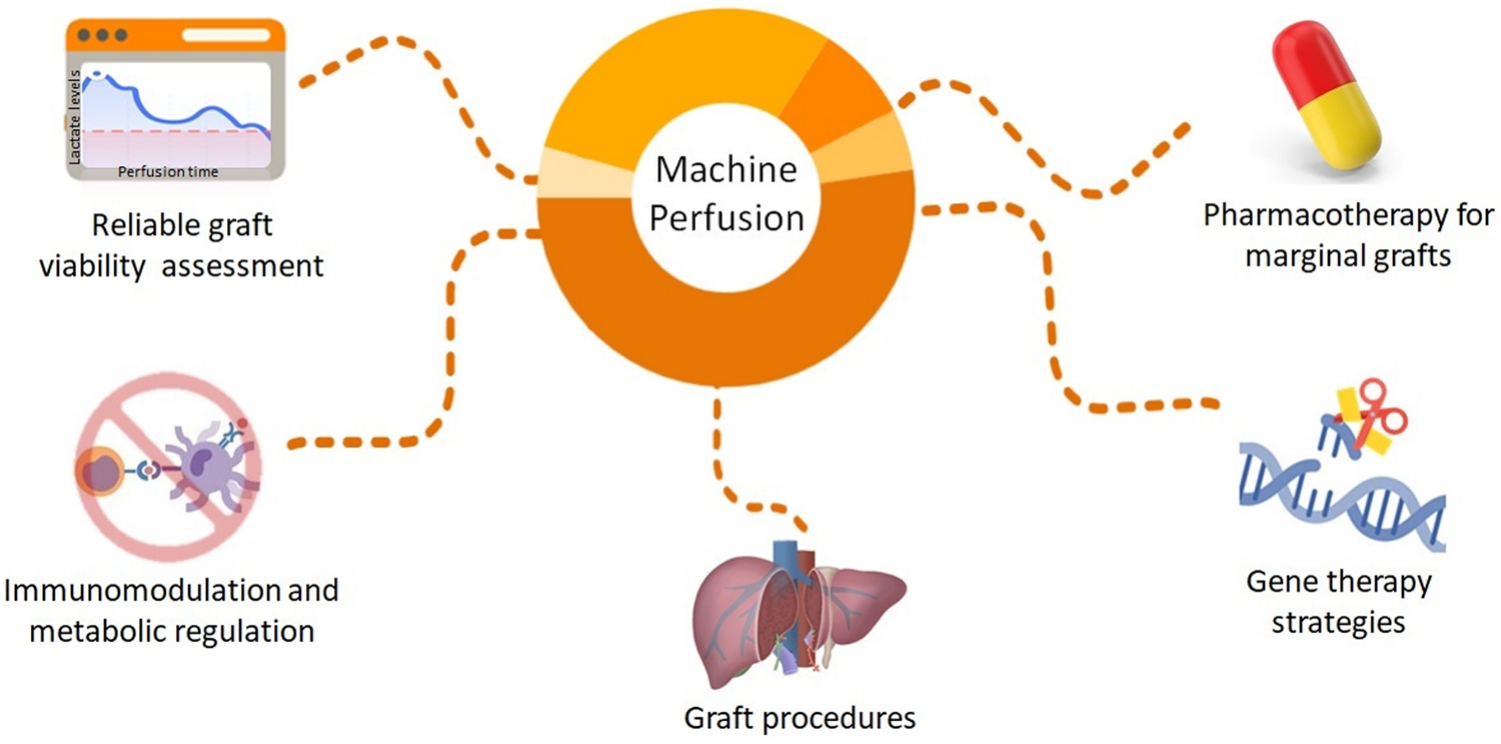
Future directions to improve organ quality by graft modification during machine perfusion of liver grafts.

Different MP systems are currently under consideration for LT. Most of them are not yet fully approved and are still undergoing experimental and clinical trials. Dynamic preservation techniques aim to provide a more supervised environment to the graft when compared to SCS. It involves perfusion of the graft under specific circumstances, including controlled temperature, perfusion flow, and special perfusate (25–27). Currently, the two types of MP most studied for use in liver transplantation are hypothermic (HMP) and normothermic (NMP). In the former, as the name implies, preservation occurs under temperatures of approximately 0–8°C, while the latter aims to provide a more physiological environment, with preservation under temperatures of approximately 35–38°C. In addition, different types of perfusate are available as preservation solutions. Common perfusates used during machine perfusion usually consist of enriched solutions—with electrolytes, buffer components, antioxidants, and amino acids among others—that facilitate organ preservation and nutrient delivery to the organs. Common solutions used for preservation in hypothermic/subnormothermic devices include the Euro-Collins solution, University of Wisconsin (UW) solution, and Histidine-tryptophan-ketoglutarate (HTK) solution (25). During NMP, solutions that provide a more physiologic environment are needed. Therefore, whole blood or blood-based solutions, such as the Steen solution or Organ Care System perfusate, are commonly used. Detailed components and descriptions of perfusate solutions are vastly described elsewhere (25). The preservation time varies, usually <12 h, with the longest viable time to date recently achieved by Clavien et al., who performed a successful transplantation of a patient whose implanted graft spent >3 days in *ex situ* preservation using NMP (28). Additional types and important considerations of each are elucidated in Table 1. Although evidence is still scarce, organ preservation under such controlled environments have opened the door for unlimited potential in graft modification and the assessment before LT (Figure 1). Important modalities are discussed below.

**Table 1:** Main characteristics of available dynamic perfusion systems.

Technique	Temperature (°C)^a^	Goal	Notes
HMP	0–8	Maintain tissue energy by enabling ATP generation through a continuous supply of metabolic substrates	Does not provide supplemental oxygen in perfusate
HOPE	0–12	HMP principle, additionally minimizing ischemia/reperfusion injury from hypoxia and mitochondrial energy depletion through continuous oxygenation	Perfusion via portal vein only. Less challenging that D-HOPE due to single vessel cannulation
D-HOPE	0–12		Perfusion via portal vein and hepatic artery. Risk of hepatic artery damage during cannulation. Additional benefits and superiority of arterial perfusion vs. perfusion via portal vein only are under current evaluation
NMP	35–38 (physiologic temperature)	Mimic physiological environment of graft to maintain metabolic activity and viability	Provide oxygen and essential substrates through perfusate. Allows graft viability assessment
SNMP	20–34 (sub-physiologic temperature)	Combine aspects of both HMP and NMP—reduce metabolic activity via hypothermic temperatures for cytoprotective benefits (HMP) but maintain a level enough for organ repair/reconditioning (NMP)	Promising strategy. More studies are needed to elucidate clinical benefit of SNMP, as well as best preservation solution while SNMP
COR	8–20	Perfusate temperature is gradually increased from HMP to SNMP levels to reduce organ injury due to sudden temperature changes upon reperfusion	Recently described strategy. More studies reflecting experience with COR for human liver perfusion are needed

ATP, adenosine triphosphate; COR, controlled oxygenated rewarming; D-HOPE, dual hypothermic oxygenated perfusion; HMP, hypothermic machine perfusion; HOPE, hypothermic oxygenated perfusion; NMP, normothermic machine perfusion; SNMP, subnormothermic machine perfusion.

^a^ Temperature range varies within devices/studies. Temperature values presented here reflect the most common range.

## Graft modification: possibilities beyond the horizon

Almost 10% of the livers recovered for transplant are not transplanted in the United States (29). Evidence shows that livers from DCD are far more likely to be discarded when compared to grafts from DBD (26.6% vs. 7.1%, respectively) (29). This comes as no surprise, taking into account the positive correlation between the prolonged warm ischemia experienced by these grafts and the detrimental effects on the graft leading to poor graft function, biliary complications, ischemic cholangiopathy, and worst graft survival (20, 30). Nevertheless, increasing reports suggest that outcomes after LT with DCD may be comparable to DBD when preservation is aided by machine perfusion (20). Therefore, the discard rate of these grafts could be further reduced, allowing a new resource to supply the donor pool.

Although DCD accounts for an important portion of liver discard, multiple other conditions affect graft usage. With the increasing obesity epidemic, macrosteatosis represents another important factor. In addition, livers with a prolonged cold ischemia time and those from donors with positive hepatitis C or B virus are also commonly discarded (31). Moreover, the organ shortage has forced transplant teams to be more flexible when it comes to selection criteria, risking postoperative outcomes when using these types of grafts. Therefore, research efforts are toward effective methods that provide treatment or amelioration of such aggravates (Table 2).

**Table 2:** Available literature related to liver graft modification using machine perfusion according to graft modification type.

Authors	Liver model type	Agent/therapy used	Study group (*n*)	Control group (*n*)	Main findings/conclusions
Immunomodulation					
Xu et al. (23)	Rat	OKY-046 (OKY) -specific thromboxane A2 synthase inhibitor	HMP-OKY (*n* = 4)	Isolated livers without preservation (*n* = 9), SCS (*n* = 9), HMP (*n* = 6)	Inhibition of TXA2 synthesis during preservation and reperfusion protects liver hepatocytes and sinusoidal endothelial cells from injuries caused by prolonged HMP
Goldaracena et al. (33)	Porcine	Anti-inflammatory strategies (alprostadil, n-acetylcysteine, carbon monoxide, sevoflurane, subnormothermic temperature)	NMP w/anti-inflammatory strategies (*n* = 5)	NMP w/o anti-inflammatory strategies (*n* = 5), SCS (*n* = 5)	The study group had reduced IL6, TNF*α*, galactosidase levels and increased IL10 levels. Providing anti-inflammatory signaling during *ex vivo* liver perfusion further improves post-transplant outcomes
Echeverri et al. (34)	Porcine	BQ123, epoprostenol, verapamil (vasodilators)	NMP w/BQ123 (*n* = 5), NMP w/epoprostenol (*n* = 5), NMP w/verapamil (*n* = 5)	NMP w/o vasodilator (*n* = 5), SCS (*n* = 5)	Livers perfused with BQ123 and verapamil had higher hepatic artery flow and reduced hepatocyte injury during perfusion compared with epoprostenol. Hepatic artery flow is significantly reduced in the absence of vasodilators during NMP
Yu et al. (35)	Porcine	MCC950 (NLRP3 inflammasome inhibitor)	HMP w/mcc950 in perfusate and administered after transplantation (*n* = 6)	HMP w/mcc950 administered after transplantation (*n* = 6), HMP w/o mcc950 (*n* = 6)	The outcome of DCD organs can be improved by the addition of MCC950 to the perfusate of HMP system, and intravenous injection of MCCC950 after transplantation
Schlegel et al. (36)	Rat	Supplemented succinate and/or mitochondrial inhibitors	Main analysis: NMP and HOPE w/succinate, malonate, rotenone, cyanide	Belzer MPS without succinate	Perfusate flavin-mononucleotide was 3–8-fold lower under hypothermic conditions. Hypothermic oxygenated perfusion restores mitochondrial function and allows viability assessment of liver grafts before implantation via measuring levels of flavin and NAD/NADH in liver perfusate
Gene therapy					
Rigo et al. (40)	Rat	HLSC-EV	NMP w/HLSC-EV (*n* = 9)	NMP w/o HLSC-EV (*n* = 10)	HLSC-EV treatment, even in a short-duration model, was feasible and effectively reduced liver injury during hypoxic NMP
Chin et al. (41)	Rat	Rat fibroblasts genetically modified with self-inactivating lentiviral vectors	NMP	Direct cell injection	Cells delivered via NMP perfusate had more even distribution in the grafts vs. those injected directly. Local cell sensors can also provide a way of monitoring organ quality as release criteria before transplant for the resuscitation of marginal donor organs
Laing et al. (43)	Human (ECD)	MAPCs	NMP hepatic artery (*n* = 3), NMP portal vein (*n* = 3)	n/a	Cells can be delivered directly to the target organ without compromising the perfusion, MAPC cells secrete a host of soluble factors with anti-inflammatory and immunomodulatory benefits
Bonaccorsi-Rian et al. (42)	Rat	Apoptosis-associated gene (FAS) using a siRNA	HOPE (4 h SCS + 1 h HOPE) (*n* = 6). HOPE (4 h SCS + 1 h HOPE) + FAS-siRNA (*n* = 6)	SCS (22 h) + PBS (*n* = 11). SCS (22 h) + FAS-siRNA (*n* = 12)	Positive effect of FAS-siRNA therapy using a potentially replicable SCS experimental protocol was observed. However, the effect of gene modulation during MP was not proven. Improved absorption of siRNA was observed during HOPE and could be favorable under particular settings with marginal grafts
Pharmacotherapy					
Nagrath et al. (46)	Rat	Amino acids, visfatin, forskolin, nuclear receptor ligands	NMP w/defatting cocktail (*n* = 7)	NMP w/o defatting cocktail (*n* = 5)	TG content reduced 65% after 3 h (compared to 30% in control). A cocktail of defatting agents can be used to rapidly clear excess lipid storage in ECD fatty livers
Liu et al. (49)	Rat	Perfusate supplemented with six defatting agents: Forskolin, GW7647, scoparone, hypericin, visfatin, GW501516	SNMP w/defatting cocktail (*n* = 5)	SNMP w/o defatting cocktail (*n* = 5)	Addition of defatting agents to the perfusate during SNMP failed to provide a strong effect in lipid reduction. Subnormothermic conditions are not ideal for defatting steatotic grafts
Taba Taba Vakili et al. (47)	Rat	GDNF	NMP w/GDNF (*n* = 4)	NMP w/defatting cocktail (*n* = 4), NMP w/vehicle (*n* = 4), non-perfused (*n* = 8)	GDNF can decrease mice liver fat content to an acceptable range and could be a potential defatting agent before liver transplantation
Goldaracena et al. (52)	Porcine	Miravirsen (HCV)	NMP (*n* = 5)	SCS (*n* = 5)	Miravirsen delivery during NMP is a potential strategy to prevent HCV reinfection after liver transplantation
Boteon et al. (50)	Human (ECD)	Forskolin, hypericin, scoparone, visfatin, GW501516, L-carnitine	NMP w/defatting cocktail (*n* = 5)	NMP w/o defatting cocktail (*n* = 5)	Pharmacological modulation of lipid metabolism during NMP can promote quick defatting of steatotic livers (reduced tissue TG by 38% and macrovesicular steatosis by 40%) and improve functional recovery, decrease vascular resistance, and reduce reperfusion injury
Xu et al. (48)	Rat	Yarmush formula (Y), 2 polyphenols, epigallocatechin-3-gallate, resveratrol (Y'-GW + E + R)	NMP w/Y'-GW + E + R (*n* = 5)	NMP w/Y (*n* = 5), NMP w/Y + E + R (*n* = 5), NMP (*n* = 4)	Superiority of novel Y'-GW + E + R in terms of safety (decreased carcinogenic potential) and reducing hepatotoxicity

ALT, alanine aminotransferase; AMP, adenosine monophosphate; AST, aspartate transaminase; ATP, adenosine triphosphate; BQ123, endothelin1 antagonist; COR, controlled oxygenated rewarming; DCD, donors after circulatory death; D-HOPE, dual hypothermic oxygenated perfusion; ECD, extended criteria donor; FAS-siRNA, apoptosis-associated gene using a small interfering RNA; GDNF, glial cell line-derived neurotrophic factor; HCV, hepatitis C virus; HLSC-EV, human liver stem cells-derived extracellular vesicles; HMP, hypothermic machine perfusion; HO-1/BMMSC, bone marrow mesenchymal stem cells modified with heme oxygenase 1 gene; HOPE, hypothermic oxygenated perfusion; IL, interleukin; INR, international normalized ratio; MAPC, multipotent adult progenitor cells, MPS, machine perfusion system; NAD, nicotinamide adenine dinucleotide; NADH, nicotinamide adenine dinucleotide (NAD) + hydrogen (H); NLRP3, NOD-like receptor protein-3; NMP, normothermic machine perfusion; RNA, ribonucleic acid; SCS, static cold storage; SNMP, subnormothermic machine perfusion; TG, triglycerides; TNFα, tumor necrosis factor; TXA2, thromboxane A2; TXB2, thromboxane B2.

### Immunomodulation and metabolic regulation of the liver while on MP

The intense immunologic response triggered by hepatic ischemia denotes a threat for graft viability and transplant success. Therefore, dampening known inflammatory mediators of ischemic injury is a promising strategy (32). In addition, the opposite remains true, and upregulating anti-inflammatory pathways may also play an important role (32). Goldaracena et al. demonstrated improved endothelial cell function and graft viability after the addition of prostaglandin E1, n-acetylcysteine, carbon monoxide, and sevoflurane in an *ex vivo* subnormothermic model using porcine livers (33). These additives were considered due to their anti-inflammatory, cytoprotective, and vasodilator properties (33). A reduction in hepatocyte injury and inflammatory mediators (IL-6, tumor necrosis factor *α*) has also been found after treatment with vasodilator agents, particularly with endothelin 1 antagonist BQ123 and verapamil during NMP in porcine models (34). Additional strategies further support the use of MP as a promising tool to deliver immune-specific therapies (35).

In addition to targeting immunologic pathways, metabolic-specific strategies have also demonstrated great potential. Previous experimental studies on animal models support this idea. Xu et al. (23) added a specific thromboxane A2 synthase inhibitor to the perfusate during HMP. The authors found that grafts in the study group demonstrated reduced portal pressure during reperfusion, lower lactate dehydrogenase levels in the perfusate, and significant improvement in tissue edema and hepatocellular necrosis (23). Recently, Schlegel et al. implemented their animal model to a clinical setting and evaluated mitochondrial metabolism (36). The authors added succinate and mitochondrial inhibitors during HOPE and NMP of DCD rat liver. Their findings suggest that the HOPE treatment provides mitochondrial protection from ischemic injury in a higher degree than normothermic condition, and even allows graft viability assessment pre-transplantation (36).

Although there are currently not enough data to determine which particular strategy contributes the most to the beneficial effect of pre-treatment with immunomodulatory agents during machine perfusion, these preliminary results highlight the importance of continuous research toward modulation of biochemical pathways involved in cell injury to improve organ preservation.

### Gene therapy strategies

Through the modification of gene expression by supplementation, silencing, or repair, gene therapy can treat, cure, or prevent conditions that would have been otherwise intractable (37, 38). Currently, the research on liver-directed gene therapies focuses on inherited metabolic liver diseases and bleeding disorders (38). However, little is known about the role of gene therapy for liver transplantation using machine perfusion. Increasing research interest have focused on gene silencing techniques with small RNA interference and the use of mesenchymal stem cells (MSCs) added to the perfusate during MP (39). In addition, novel strategies, including stem cell-derived extracellular vesicles, have shown promising results inspiring future research in the field (40).

The current methods employed for gene therapy include *ex vivo* administration, with cell modification outside the patient, or *in vivo*, via systemic or local administration using carrier vectors—generally non-viral or viral vectors (38). Hence, specific targeting to the desired organ is limited, jeopardizing the expected effects and exposing it to processes of clearance, filtration, and enzymatic breakdown in the serum and different organs (39, 41). To address this limitation, Chin et al. developed a model using engineered rat fibroblasts infused into the livers of rats while undergoing NMP to simulate *in vivo* conditions (41). Bioluminescence assays demonstrated the superiority of the continuous flow-based seeding process, by using NMP, to obtain adequate distribution of the engineered fibroblasts within the liver tissue when compared to a simple injection (41). In addition, the authors found no negative impact on graft survival and function after transplant when compared with control grafts. This innovative study laid the foundation for the implementation of MP as a feasible delivery method for targeted therapies. Another recent experimental study using HOPE as a delivery system demonstrated increased absorption of small interfering RNA compounds during perfusion (42). Unfortunately, no strong conclusions were drawn in terms of the benefit of the used compounds themselves (42). Laing et al. further validated the role of MP as a tool to provide direct and targeted organ delivery of cellular therapy in a human model of liver transplantation under normothermic perfusion conditions (43). The available experimental experiences provide proof-of-concept and indicate that merging MP with gene therapy is feasible. However, further studies are needed to determine if there is a true clinical benefit of combining these two complex modalities.

Similarly, the delivery of MSC via MP offers the possibility of taking advantage of its immunomodulatory and regenerative effects, without the burden of conventional MSC therapy associated risks. MSC therapy is associated with prothrombotic activity, leading to microvascular thrombosis and pulmonary embolism, as well as increased lung uptake/entrapment, reducing its delivery to other organ targets (44). Nevertheless, further studies to validate the benefits of MP for this particular use are needed.

Although novel in the liver transplant field, a combination of gene therapy and machine perfusion preservation has the potential to offer therapeutic options for graft reconditioning and rejection prevention. However, further research is needed to determine the true benefits of this novel modality. In addition, the evaluation of cost-effective benefits of these therapies is warranted as, currently, gene therapy alone can represent significant expenses, limiting patient access and increasing healthcare-associated expenses (45).

### Pharmacotherapy for marginal grafts

#### Steatotic grafts

Steatotic grafts continue to increase among the donor pool and constitute one of the major reasons for graft discard. Therefore, the reduction of fat content in the graft before transplantation seems like a long-needed strategy. Nagrath et al. first proved this concept in 2009 (46). Liver grafts from hepatocellular steatotic-induced rats were perfused for 3 h using NMP. Perfusion was carried out with supplemented perfusate with a defatting cocktail composed of multiple agents with an individually known stimulatory effect on triglyceride secretion and/or β-oxidation (46). The authors found that lipid clearance was significantly higher in grafts perfused with the defatting cocktail when compared to the control perfusate. After 3 h of perfusion, the triglyceride content decreased by 65% in the study group, but only by 30% in the control group. Important histologic changes were also observed, particularly in the periportal region, including a reduction of lipid vesicles in hepatocytes, restored hepatocellular volume, and increased diameter in the sinusoid diameter (46). In addition, the expression of regulatory genes involved in lipid oxidation and secretion pathways was also increased. The authors suggested that their results imply a potential option to salvage moderate to severely steatotic grafts and, therefore, increase the donor pool (46). Similar results were later achieved using animal models and different agents including Glial cell line-derived neurotrophic factor and multidrug combinations, achieving good results in the reduction of lipid content and hepatocellular damage (47, 48). Conflicting results using subnormothermic conditions have been reported (49).

The promising results evidenced during animal models were tested in human livers undergoing MP supplemented with defatting agents for the first time in 2019 (50). The group evaluated 10 discarded steatotic human liver grafts, including DCD, and grafts with different degrees of steatosis (ranging from non-to severe) perfused with a defatting cocktail and L-carnitine supplementation (50). In alignment with previous experimental reports (46), lipid enzyme upregulation was observed, along with the increased activity of lipid regulation metabolic pathways (50). Over a perfusion period of 6 h, triglycerides levels were reduced by 38% in the study group, compared with 7% in those undergoing NMP alone. In addition, the authors observed a time-dependent decrease of 40% in the macrosteatosis rate after 6 h and of 50% after 12 h of perfusion in the treatment group, with no changes in the control group. Importantly, a statistically significant difference was found in terms of organ viability. While all the treated organs reached the viability criteria for transplantation, only two in the control group did (50). These available promising results, obtained by lipid metabolic reconditioning of steatotic grafts while undergoing machine perfusion, could reshape the way organ allocation and procurement are performed. Further studies are needed to determine the best defatting agent combination to use, as well as to better elucidate short- and long-term effects of this novel strategy in the clinical setting.

#### Hepatitis C-positive grafts

Since the approval of direct acting antiviral agents effective against hepatitis C virus (HCV), the acceptance of grafts from viremic donors for transplant into negative recipients have been less stringent (51). This allows more availability of grafts for transplantation for some recipients. However, the treatment regimen after LT with a HCV-positive graft is usually long—approximately 12 weeks—incurring high costs, ongoing surveillance, and possible drug side effects and drug-to-drug interactions (51). Therefore, the reduction or complete eradication of the graft viral load before transplantation would be an ideal scenario.

On the other hand, transplantation of HCV-negative grafts into a positive recipient results in immediate reinfection of the graft, with a more aggressive presentation and important rate of cirrhosis recurrence within 5 years (52). Therefore, pre-treatment of HCV-negative grafts before transplantation into a positive recipient is a promising preventive strategy. Goldaracena et al. first explored this concept in their experimental porcine model (52). The authors measured the impact of Miravirsen on HCV replication in grafts undergoing MP preservation. Miravirsen is an antiviral agent that targets microRNA-122, an important component in the replication of HCV (52). It requires active metabolism for an adequate uptake; therefore, the authors used NMP for its delivery into the graft. Following a series of analyses, it was found that NMP resulted in significantly higher absorption of the agent when compared to SCS, with higher concentrations found at 2 h and 72 h after LT (52). In addition, sequestration of microRNA-122 was significantly higher among the study group. Therefore, the authors suggested that their results indicate temporary gene silencing and, possibly, a reduction in HCV replication after LT with pre-treated grafts in NMP (52). This proof-of-concept supports the necessity of an active metabolism to successfully deliver a drug *ex vivo* and allow it to achieve its actions on a specific target organ, goals that were facilitated by the environment provided by NMP. Therefore, pharmacotherapy studies—not only for HCV but also for a plethora of conditions—should focus on the use of NMP, as hypothermic conditions assert the opposite effect on metabolism.

Much is still needed to explore pharmacotherapy during MP of marginal grafts in the clinical setting. However, favorable results achieved during animal models support the notion of combining dynamic flow preservation and existing pharmacological agents to improve the quality of marginal organs beyond the benefits of dynamic flow alone. In addition to potentially serving as a drug-delivery platform, pharmacotherapy using MP allows for the detailed pharmacokinetic and pharmacodynamic assessment in human livers (53). Research teams are encouraged to continue expanding the options that these technologies have to offer. Important areas to explore the role of MP include toxicology assessment, oncologic therapies, delivery of nanoparticles, and surgical training (32, 54). Scientific, technologic, and logistic advances make graft modification using MP a tangible treatment possibility in the near future.

## Conclusion

Organ preservation using the MP system offers a vast array of possibilities for graft modification when combined with alternative therapies, including immunomodulation, gene therapy, and pharmacologic strategies. The available reports, mostly on experimental phases, are promising. However, further research on clinical settings is needed to better elucidate the true effect of graft modification pre-transplant on short- and long-term grafts and patient survival. To date, there are not enough data to support one MP device over the other for graft modification. However, the selection of hypothermic, normothermic, or subnormothermic conditions should be goal-driven based on the adjuvant therapy to be used. There is a long road ahead to develop guidelines and approve these novel therapies. However, the path looks promising.

## Data Availability

The original contributions presented in the study are included in the article/**Supplementary Material**, further inquiries can be directed to the corresponding author.
