# Supplementary material for: Comprehensive review of the application of MP and the potential for graft modification
Source: Front Transplant. 2023 May 16;2:1163539. doi: 10.3389/frtra.2023.1163539 (PMC11235300; doi:10.3389/frtra.2023.1163539)
Supplement: Supplementary file 1 [file Datasheet1.pdf]

Comprehensive review of the application of MP and potential for Graft modification

Supplemental Material

Paola A. Vargas, MD[1], Christine Yu, B.A[2], Nicolas Goldaracena, MD[1]*

[1] Division of Transplant Surgery, Department of Surgery, University of Virginia Health System, Charlottesville, Virginia, USA

[2] University of Virginia, Charlottesville, Virginia, USA

*** Correspondence:**

Nicolas Goldaracena, MD.
Department of Surgery, Division of Transplantation, University of Virginia Health System
PO Box 800709. 1215 Lee Street. Charlottesville, VA 22908-0709
Office: 434.924.9462. Fax: 434.924.5539
Email: n.goldaracena@virginia.edu

Keywords: machine perfusion, ex-vivo, liver transplantation, marginal organs, donor pool.

Type of MP	Authors	Publication year	Study period	Type of study	Study group (n)	Control group (n)	Type of graft/donors	Main findings/Conclusions
Hypothermic machine perfusion (HMP)	Guarrera et al. (5)	2010	2004-2008	Prospective cohort pilot study	HMP (n=20)	SCS (n=20)	DBD	HMP is safe, may improve graft function and attenuates classical biochemical markers of liver preservation injury.
	Guarrera et al. (55)	2011	2004-2008	Non-randomized clinical trial	HMP (n=3)	SCS (n=3)	DBD	HMP significantly reduced pro-inflammatory cytokine expression compared with SCS controls.
	Henry et al. (10)	2012	2004-2008	Non-randomized clinical trial	HMP (n=18)	SCS (n=15)	ECD	Overall reduction in the level of ischemic injury in HMP-treated liver grafts likely leading to increased safe utilization of more ECD livers and improve patient outcomes after liver transplantation.
	Guarrera et al. (11)	2015	2007-2012	Non-randomized clinical trial	HMP (n=31)	SCS (n=30)	ECD	HMP is an effective method for reducing the incidence of preservation injury.
hypothermic oxygenated perfusion (HOPE)	Dutkowski et al. (12)	2015	2012-2014 (HOPE), 2005-2014 (SCS)	Matched case analysis	HOPE (n=25)	SCS (n=50)	DCD	HOPE offers important benefits in preserving higher-risk DCD liver grafts
	Schlegel et al. (15)	2019	2012-2017	Retrospective cohort study	HOPE (n=50)	SCS (n=100)	DCD (study group, control group n=50), DBD (control group n=50)	Simple end-ischemic perfusion approach is effective and may open the field for safe utilization of extended DCD liver grafts.
	Ravaioli et al. (56)	2020	2016-2017	Matched case clinical trial	HOPE (n=10)	SCS (n=30)	ECD	HOPE is a safe and effective system to reduce ischemic injury and improve graft function.
	Czigany et al. (9)	2021	2017-2020	Multicenter RCT	HOPE (n=23)	SCS (n=23)	ECD	Superiority of HOPE for reduction of early allograft injury and improvement of post-transplant outcomes in ECD-DBD liver transplantation.
Dual hypothermic oxygenated perfusion (D-HOPE)	Van Rijn et al. (13)	2017	2014-2015	Prospective case-control study	DHOPE (n=10)	SCS (n=20)	DCD (study group), DBD (control group)	DHOPE restores hepatic ATP, reduces reperfusion injury, and is safe and feasible.

	Van Rijn et al. (14)	2018	2014-2015	RCT	DHOPE (n=10)	SCS (n=20)	DCD (study group), DBD (control group)	DHOPE have the potential to preserve regenerative capacity of donor bile ducts.
	Burlage et al. (16)	2018		Non-randomized clinical trial	DHOPE (n=10)	SCS (n=9)	DCD	Reperfusion of a DHOPE-preserved liver may lead to a decrease in blood potassium concentrations.
	Van Rijn et al. (57)	2021	2016-2019	Multicenter RCT	DHOPE (n=78)	SCS (n=78)	DCD	Hypothermic oxygenated machine perfusion led to a lower risk of non-anastomotic biliary strictures following the transplantation of livers obtained from DCD than conventional SCS.
Normothermic machine perfusion (NMP)	Ravikumar et al. (58)	2016	2011-2013	Non-randomized prospective clinical trial	NMP (n=20)	SCS (n=40)	DBD (n=16), DCD (n=4)	NMP showed decreased EAD rate in treatment group, comparable hospital stay length, 100% 30-day recipient and graft survival. NMP has potential for enhanced hepatic protection.
	Bral et al. (59)	2017	2015	Non-randomized pilot study	NMP (n=10)	SCS (n=30)	DBD (n=6), DCD (n=4)	Equivalent 30-day patient survival and 6-month graft survival. NMP in a North American setting is feasible.
	Selzner et al. (60)	2016	2015	Non-blinded pilot study	NMP (n=10)	SCS (n=30)	DCD	NMP with Steen solution as perfusate is safe for LT.
	Nasralla et al. (61)	2018	2014-2016	RCT	NMP (n=121)	SCS (n=101)	DBD, DCD	NMP significant reductions in peak AST and EAD rates. Therefore, can safely extend preservation times and increase organ utilization without compromising outcomes.
	Ghinolfi et al. (62)	2018	2016-2018	RCT	NMP (n=10)	SCS (n=10)	DCD	NMP with older liver grafts is associated with histological evidence of reduced ischemic injury, although the clinical benefit remains to be demonstrated.
	Mergental et al. (63)	2020	2016-2018	Prospective non-randomized trial	NMP (n=22)	SCS (n=22)	DBD, DCD	NMP allows for objective assessment of high-risk organs and successful transplantation of currently unutilized livers without any incidence of primary non-function

	Markmann et al. (64)	2022	2016-2019	Multicenter RCT	NMP (n=151)	SCS (n=142)	DBD, DCD	NMP demonstrates potential for superior post-transplant outcomes and increased donor liver use.
	Quintini et al. (65)	2022	2020-2021	Non-randomized prospective clinical trial	NMP (n=15)	n/a	DCD (n=11), DBD (n=4)	Grafts deemed non-transplantable using SCS can be successfully assessed and transplanted. Current machine viability criteria may be too restrictive.
Other/ Miscellaneous	Van Leeuwen et al. (17)	2019	2017-2019	Non-randomized clinical trial	DHOPE-COR-NMP (n=11)	SCS (n=60)	DBD (n=36), DCD (n=24)	No statistically significant difference in patient survival at 3, 6, and 12 months post transplantation between the three groups

Abbreviations: ALT - alanine aminotransferase; AST - aspartate aminotransferase; COR - controlled oxygenated rewarming; DBD - donation after brain death; DCD - donation after circulatory death; EAD - early allograph dysfunction; ECD - extended criteria donor; FFP - fresh frozen plasma; INR - international normalized ratio; LFT - liver function test; NMP- normothermic machine perfusion; PRS - post reperfusion syndrome; SCS - static cold storage.
